# Evaluation of electrohysterogram measured from different gestational weeks for recognizing preterm delivery: a preliminary study using random Forest

**DOI:** 10.1016/j.bbe.2019.12.003

**Published:** 2020

**Authors:** Jin Peng, Dongmei Hao, Lin Yang, Mengqing Du, Xiaoxiao Song, Hongqing Jiang, Yunhan Zhang, Dingchang Zheng

**Affiliations:** a College of Life Science and Bioengineering, Beijing University of Technology, Intelligent Physiological Measurement and Clinical Translation, Beijing International Platform for Scientific and Technological Cooperation, Beijing, China; b Beijing Haidian Maternal and Children Health Hospital, Beijing, China; c Centre for Intelligent Healthcare, Faculty of Health and Life Science, Coventry University, Coventry, UK

**Keywords:** EHG, electrohysterogram, RF, random forest, PE, preterm delivery before the 26^th^ week of gestation, PL, preterm delivery after the 26^th^ week of gestation, TE, term delivery before the 26^th^ week of gestation, TL, term delivery after the 26^th^ week of gestation, IUPC, intrauterine pressure catheter, TOCO, tocodynamometer, K-NN, K-nearest, LDA, linear discriminant analysis, QDA, quadratic discriminant analysis, SVM, support vector machine, ANN, artificial neural network, DT, decision tree, TPEHG, term-preterm electrohysterogram, RMS, root mean square, *τ*_z_, zero-crossing, PF, peak frequency, MDF, median frequency, MNF, mean frequency, SE, energy values in signal, SM, maximum values in signal, SS, singular values in signal, SV, variance values in signal, AR, auto-regressive model, Tr, time reversibility, CorrDim, correlation dimension, SampEn, sample entropy, LE, Lyapunov exponent, SD, standard deviation, ADASYN, adaptive synthetic sampling approach, ACC, accuracy, AUC, the area under the curve, ROC, the receiver operating characteristic curve, Electrohysterogram (EHG), Feature extraction, Gestational week, Preterm delivery, Random forest (RF).

## Abstract

Developing a computational method for recognizing preterm delivery is important for timely diagnosis and treatment of preterm delivery. The main aim of this study was to evaluate electrohysterogram (EHG) signals recorded at different gestational weeks for recognizing the preterm delivery using random forest (RF). EHG signals from 300 pregnant women were divided into two groups depending on when the signals were recorded: i) preterm and term delivery with EHG recorded before the 26^th^ week of gestation (denoted by PE and TE group), and ii) preterm and term delivery with EHG recorded during or after the 26^th^ week of gestation (denoted by PL and TL group). 31 linear features and nonlinear features were derived from each EHG signal, and then compared comprehensively within PE and TE group, and PL and TL group. After employing the adaptive synthetic sampling approach and six-fold cross-validation, the accuracy (ACC), sensitivity, specificity and area under the curve (AUC) were applied to evaluate RF classification. For PL and TL group, RF achieved the ACC of 0.93, sensitivity of 0.89, specificity of 0.97, and AUC of 0.80. Similarly, their corresponding values were 0.92, 0.88, 0.96 and 0.88 for PE and TE group, indicating that RF could be used to recognize preterm delivery effectively with EHG signals recorded before the 26^th^ week of gestation.

## 1 Introduction

Preterm delivery, defined as birth before 37 completed weeks of gestation, is a leading cause of neonatal morbidity and mortality, and has long-term adverse consequences for fetal health [1]. Accurate diagnosis of preterm delivery is one of the most significant problems faced by obstetricians.

The existing measurement techniques for diagnosing preterm delivery include tocodynamometer (TOCO), ultrasound and fetal fibronectin. However, they are subjective, or suffer from high measurement variability and inaccurate diagnosis or prediction of preterm delivery [2]. TOCO is often influenced by sensor position, the tightness of binding by the examiner and maternal movement. Short cervical length measured by transvaginal ultrasonography has been associated with an increased risk of preterm delivery. But its accuracy for prediction of preterm delivery is not satisfied due to the high false positive rate. Fetal fibronectin test, which is performed like a pap smear, has not been shown to accurately predict preterm delivery in women who are at low risk or who have no obvious symptoms. Comparatively, electrohysterogram (EHG) which reflects the sum of the electrical activities of the uterine cells could be recorded noninvasively from the abdominal surface. The parameters of EHG signals might provide an effective tool for the diagnosis and prediction of preterm delivery [3]. Therefore, using EHG signal is a reliable method at evaluating uterine activity and it has been used in analyzing uterine activity of non-pregnant women as well [4].

Many features have been extracted from EHG signals to recognize preterm delivery, which can be grouped into three classes: linear features, nonlinear features and features related to EHG propagation [5]. Time, frequency and time-frequency features, such as root mean square, median frequency, peak frequency and energy distribution have been used to characterize EHG signals and distinguish between term and preterm delivery [[5], [6], [7]]. Besides, nonlinear features, including correlation dimension (CorrDim) [8], sample entropy (SampEn) [9], Lyapunov exponent (LE) [10], and multivariate multiscale fuzzy entropy [11] have been applied to describe the nonlinear interactions between billions of myometrium cells [12,13]. In recent years, the propagation velocity, direction of the EHG signals, intrinsic mode functions from empirical model decomposition (EMD) [14] have been proposed as the potential discriminators to predict the progress of pregnancy. However, selection of EHG features was somehow arbitrary in these published studies. A comprehensive analysis of these feature differences between preterm and term delivery would therefore be clinically and physiologically useful.

Machine-learning algorithms have been investigated to recognize the preterm delivery using EHG signals [15]. Conventional classifiers include the K-nearest neighbors (K-NN), linear and quadratic discriminant analysis (LDA and QDA, respectively), support vector machine (SVM) [6], artificial neural network (ANN) classifiers [8,16,17], decision tree (DT) [18], penalized logistic regression, rule-based classifier [19] and stacked sparse autoencoder (SSAE) [20]. However, the K value of the K-NN classifier is set subjectively, LDA and QDA are affected by sample distribution, ANN and SSAE have high computational complexity [16], and SVM requires additional steps to reduce the dimension of the extracted features [21]. The published studies have reported that ANN, SSAE, Adaboost, DT, SVM, logistic and polynomial classifier have achieved better performance in recognizing preterm delivery. However, these classifiers were evaluated on different database using different EHG features, and therefore unable to determine the most significant features for predicting preterm delivery. Random forest (RF) is an ensemble learning method for classification. DT is the base learner in RF, which has been employed in data mining and feature selection [22]. Classification accuracy could be improved by growing an ensemble of trees and letting them vote for the most popular class. Ren et al. reported that RF with simpler structure achieved the same accuracy as ANN for classifying preterm delivery with EHG signals [17]. Idowu et al. [19] also indicated that RF performed the best and robust learning ability.

The main aim of this study was to evaluate the EHG signals recorded at different gestational weeks for recognizing preterm and term delivery using RF. Meanwhile, the importance of EHG features for predicting preterm delivery would be ranked.

## 2 Materials and methods

**Fig. 1:**
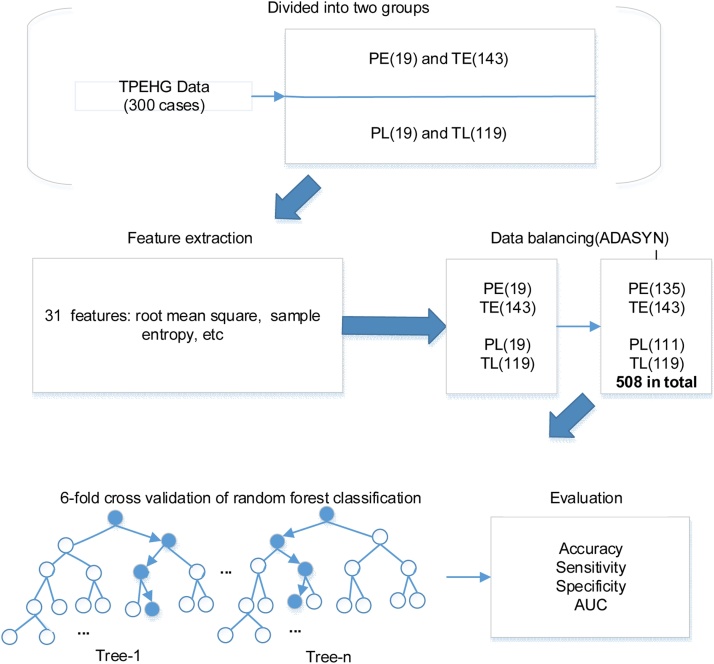
Flow chart of the proposed method. Note: PE: preterm delivery with EHG recorded before the 26^th^ gestation week, TE: term delivery with EHG recorded before the 26^th^ gestation week, PL: preterm delivery with EHG recorded after the 26^th^ gestation week, TL: term delivery with EHG recorded after the 26^th^ gestation week, ADASYN: adaptive synthetic sampling approach, AUC: the area under the curve of receiver operating characteristic.

The overview flowchart of the proposed method in this study is shown in Fig. 1. Briefly, EHG signals from 300 pregnant women were divided into two groups depending on whether the EHG signals were recorded before or after 26^th^ week of gestation. Thirty-one linear and nonlinear features were then derived from each EHG signal and fed to a RF classifier for automatic identification of term and preterm delivery, and the importance of features was ranked by DTs. The performance of RF for recognizing preterm delivery was then evaluated and compared between EHG signals recorded at different gestational weeks. The details of each step are presented in Fig. 1.

### 2.1 EHG database

**Fig. 2:**
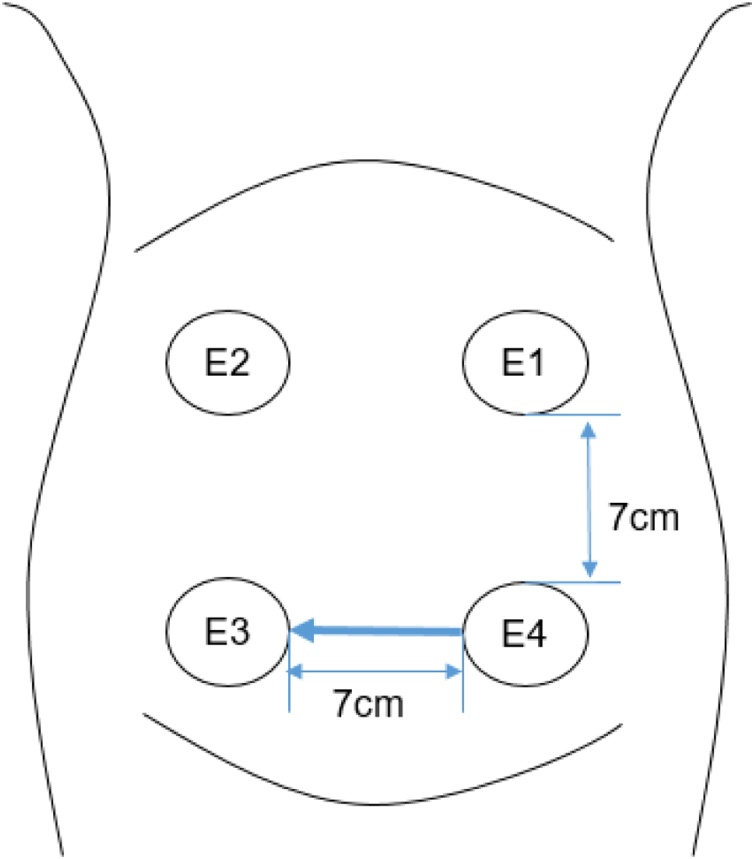
The placement of the electrodes on the abdomen. Channel 1=E2-E1, Channel 2=E2-E3, Channel 3= E4-E3

EHG signals in our study were from the open access term-preterm EHG (TPEHG) database developed in 2008 at the Faculty of Computer and Information Science, University of Ljubljana, Ljubljana [23]. Three channels of EHG signals were recorded from the abdominal surface using four electrodes, as shown in Fig. 2. Three-channel EHG signals were measured between the topmost electrodes (channel 1: E2-E1), the leftmost electrodes (channel 2: E2-E3), the lower electrodes (channel 3: E4-E3) separately. The recording time was 30 min with the sampling frequency of 20 Hz. A previously published research has confirmed that the EHG from channel 3 was regarded as the most distinguishable signals for classifying preterm and term delivery [17]. Therefore, as a pilot study, channel 3 was selected for further analysis.

**Fig. 3:**
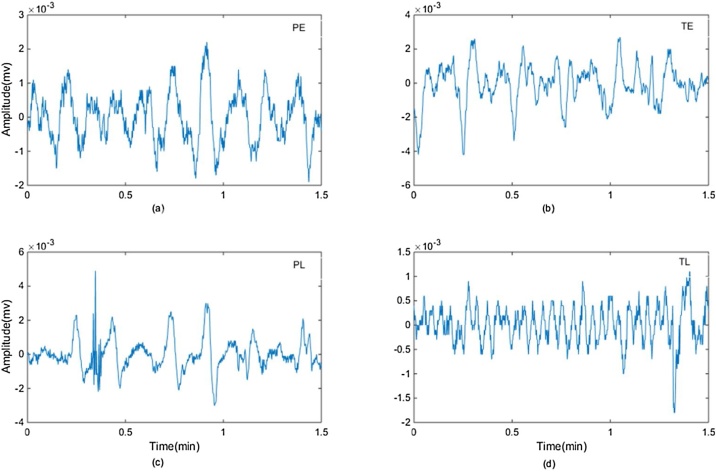
Examples of EHG segments (1.5 min) from: (a) PE (recorded before the 26^th^ week of gestation, with preterm delivery); (b) TE (recorded before the 26^th^ week of gestation, with term delivery); (c) PL (recorded during or after the 26^th^ week of gestation, with preterm delivery); (d) TL (recorded during or after the 26^th^ week of gestation, with term delivery).

EHG signals from 300 pregnant women (262 cases of term delivery, and 38 cases of preterm delivery) were divided into two groups depending on when the signals were recorded: i) preterm and term delivery with EHG recorded before the 26^th^ week of gestation (denoted by PE and TE group, 19 and 143 cases respectively), and ii) preterm and term delivery with EHG recorded during or after the 26^th^ week of gestation (denoted by PL and TL group, 19 and 119 cases respectively).Table1 shows the number of EHG recordings in PE and TE group and in PL and TL group. Fig. 3 shows four typical examples of EHG segments from each group.

**Table 1:** The number of EHG recordings in PE and TE, PL and TL groups from TPEHG database.

Recording time	Delivery time	
	<37 weeks (Preterm)	≥ 37 weeks (term)
< 26^th^ week of gestation	Preterm Early (PE, n = 19)	Term Early (TE, n = 143)
≥ 26^th^ week of gestation	Preterm Late (PL, n = 19)	Term Late (TL, n = 119)

### 2.2 EHG signal preprocessing

The main frequency component of EHG signal ranges between 0 and 5 Hz [24]. The EHG signals preprocessed by the band-pass filter of 0.08−4 Hz were selected from the TPEHG database, in which the interferences from fetal and maternal electrocardiogram, respiratory movement, motion artifacts and 50/60 Hz power noise had been removed [25]. Furthermore, the first and last 5 min of EHG segments were abandoned to avoid the transient effects due to filtering process [18], and the remaining 20 min EHG signals were used for further analysis.

### 2.3 EHG features extraction

Thirty-one features were extracted with time domain, frequency domain, time-frequency domain and nonlinear analysis as follows.

#### 2.3.1 Root mean square (RMS)

RMS is a conventional method for investigating signal amplitude changes. Given a time series of $\;x\left(i\right);i=0,\ldots ,N-1$, N is the signal length, here N = 600. RMS was calculated as:

(1) $$RMS=\sqrt{\frac{1}{N}\sum_{i=0}^{N-1}x^{2}(i)}$$

#### 2.3.2 Autocorrelation zero-crossing($\tau_{Rxx}$)

Autocorrelation zero-crossing, ${\text{τ}}_{Rxx},$ is defined as the first zero-crossing starting at the peak in the autocorrelation $R_{xx}$ (τ) of the signal x(t) [26]. Considering the data distribution, ${\text{τ}}_{Rxx}$ was calculated as:

$$\;R_{xx}\left({\text{τ}}_{Rxx}\right)=0$$

(2) $$R_{xx}\left(\text{τ}\right)=\sum_{i=1}^{N}sgn\left(-x\left(i\right)x\left(i+\text{τ}\right)\right)$$

sgnx=1,x>00,x<0

where $x\left(i\right)$ is the amplitude of EHG signal at sampling point i.

#### 2.3.3 Peak frequency (PF)

PF corresponds to the largest amplitude peak of the EHG signal power spectrum p which was calculated using the fast discrete Fourier transform of each signal. PF was calculated as follows:

(3) $$f_{max}=\text{arg}\left(\frac{f_{s}}{N}{max}_{i=0}^{N-1}P\left(i\right)\right)$$

where $f_{s}$ = 20 Hz is the sampling frequency.

#### 2.3.4 Median frequency (MDF)

MDF is defined as the frequency above where the sums of the parts above and below the frequency-power spectrum P are the same. MDF was calculated follows:

(4) $$f_{med}=i\frac{f_{s}}{N},\;\sum_{i=0}^{i}P\left(i\right)=\;\sum_{i}^{i=N-1}P(i)$$

where i is the i-th line of the power spectrum.

#### 2.3.5 Mean frequency (MNF)

MNF is the centroid frequency of the power spectrum and is defined as follows:

(5) $$\text{M}\text{N}\text{F}=\frac{\sum_{i=1}^{I}f_{i}P_{i}}{\sum_{i=1}^{I}P_{i}}$$

where $p_{i}$ is the i -th line of the power spectrum; $\;f_{i}\;$ is the frequency variable; and I is the highest harmonic ($I=\frac{N}{2})$. N is the signal length, here N = 600.

#### 2.3.6 Features extracted from wavelet decomposition

**Fig. 4:**
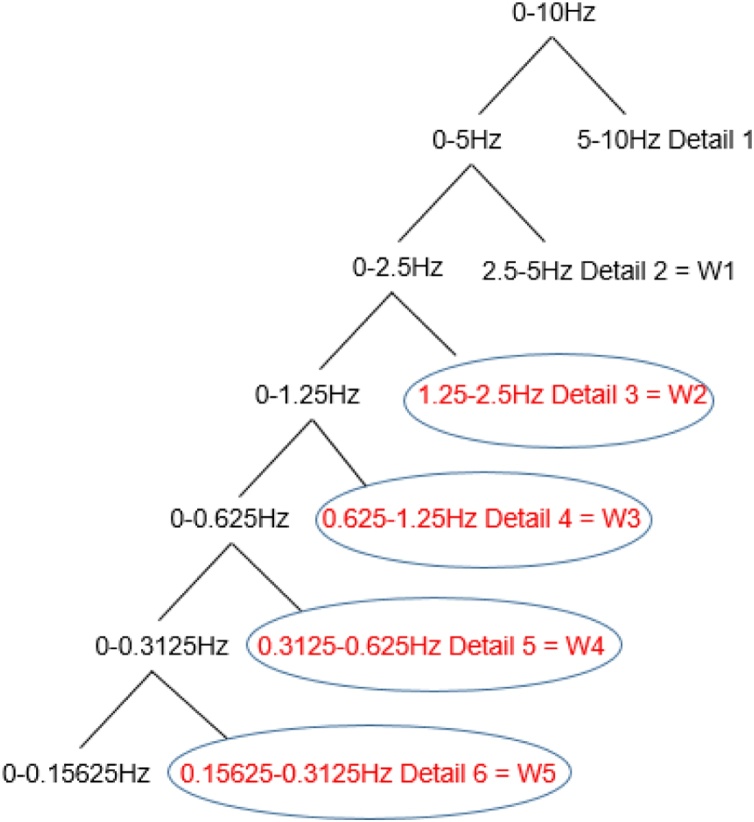
Wavelet decomposition of EHG signal.

Features from the wavelet decomposition mainly included the maximum, energy, singular and variance values. Each EHG recording was decomposed into detail coefficients with symlet 5 [26] as shown in Fig. 4. The energy ${SE}_{2},SE_{3},SE_{4},\;SE_{5}$, the maximum ${SM}_{2},SM_{3},SM_{4},SM_{5}$, the singular ${SS}_{2},SS_{3},SS_{4},SS_{5}$ and the variance ${\;SV}_{2},SV_{3},SV_{4},SV_{5}$ of the wavelet coefficients were then calculated at the detail level of: 3,4,5,6 (named W2, W3, W4 and W5 respectively).

#### 2.3.7 Features extracted from autoregressive (AR) model

AR is a time series model that uses observations from previous time steps as input to a regression equation to predict the value at the next time step.

(6) $$x_{m}=\sum_{i=1}^{p}a_{i}x_{m-i}+\varepsilon_{m}$$

where p is the order of AR model, here p = 5. *a_1_, a_2_, a_3,_ a_4,_ a_5_* and residual *e* were the model features. ε_m_ is the white noise.

##### 2.3.7.1 Time reversibility (Tr)

Tr was used to describe if the probabilistic properties of a time series are changeable with respect to time reversal. A stochastic process is defined as time-reversible if it is invariant under the reversal of the time scale [33]. Tr was calculated as follows:

(7) $$Tr(t)=(\frac{1}{M-\tau }){\sum_{m=\tau +1}^{M}(x_{m}-x_{m-\tau })}^{3}$$

Where *x* is a time series with M samples, M = 24,000(20 Hz☓60 s/min☓20 min)and τ is the time delay, here τ = 1.

#### 2.3.8 Lyapunov exponent (LE)

LE characterizes the rate of separation between adjacent tracks in the phase space. λ is a measure of how fast a trajectory converges from a given point into some other trajectory:

(8) $$\lambda =\underset{i\to \text{∞}}{\text{lim}}\frac{1}{i}\text{l}\text{n}(\frac{d_{i}}{d_{0}})$$

where $d_{0}$ represents the Euclidean distance between two states of the system at some arbitrary time i.

#### 2.3.9 Sample entropy (SampEn)

SampEn measures the irregularity of a time series of finite length. The more unpredictable the time series is, the higher its SampEn. For a given embedding dimension m, tolerance r and number of data points M, SampEn ( m, r, M) is the negative logarithm of the probability that if two sets of simultaneous data points of length m have distance<  r then the two sets of simultaneous data points of length m +1 also have distance<  r. We had the EHG time-series of length M = $\left\{x_{1,}x_{2,}{\ldots ,x}_{M}\right\}$ with a constant time interval *τ*. The number of vector pairs in template vectors of length m and m +1 were counted having d[X_m_(i), X_m_(j)] < r and denoted it by B and A respectively. The sample entropy was defined as:

(9) $$\text{S}\text{a}\text{m}\text{p}\text{E}\text{n}=-\text{log}\frac{A}{B}$$

A = number of template vector pairs having d[X_m+1_(i), X_m+1_(j)] < r of length m +1

B = number of template vector pairs having d[X_m_(i), X_m_(j)] < r of length m

m varied from 1 to 2, and r from 0.1SD to 0.25SD (SD is the standard deviation of a time series ). In this study, m = 2, r = 0.1SD, which has got promising result in other result [26].

#### 2.3.10 Correlation dimension (CorrDim)

CorrDim( $D_{corr}$ )is a measure of the dimensionality of the space occupied by a set of random points. For a time series of M points: { $y\left(i\right)$:1≤ i ≤ M }, the formula are as follows:

(10) $$D_{corr}=\underset{t\to 0}{\text{lim}}\frac{\text{log}\left(C\left(t\right)\right)}{\text{log}\left(t\right)}$$

(11) $$C\left(t\right)=\underset{M_{C}\to \text{∞}}{\text{lim}}\frac{1}{{M_{C}}^{2}}\sum_{i=1}^{M_{C}}\sum_{j=i+1}^{M_{C}}\theta \left(t-\left|y\left(i\right)-y(j)\right|\right)$$

where θ[.] is the Heaviside function, t is the limit for the distance between two points on the system trajectory, M is the number of the trajectory points. y is the EHG time series. $M_{C}$ = 23,999(*M-1, M =* 24,000).

### 2.4 Comparison of EHG features between term and preterm delivery

The mean ± SD of the derived EHG features were calculated across all the cases in the PE and TE group, and PL and TL group. Non-parametric t-test (Mann–Whitney U test) was performed using SPSS 22 (IBM Corporation, New York, United States) to assess the difference of EHG features between PE and TE, and between PL and TL. A p-value below 0.05 was considered statistically significant.

### 2.5 Term and preterm classification

#### 2.5.1 Adaptive synthetic sampling approach (ADASYN)

**Fig. 5:**
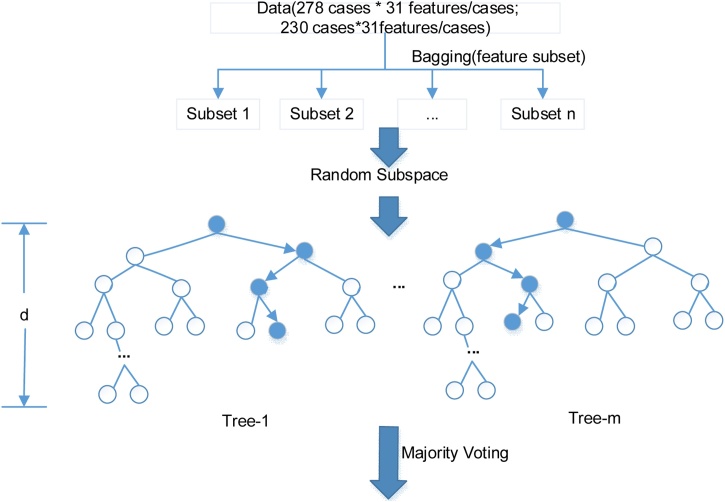
The schematic diagram of RF classifier. Note: The maximum number of features in each subset is 29, the number of DT m = 89 and the depth of each tree d = 20. Two RF classifiers were developed, respectively, one of which using 278 cases from PE and TE group and the other using 230 cases from PL and TL group.

TPEHG dataset is not balanced in term of the sample size between term delivery (majority class, 262 cases) and preterm deliveries (minority class, 38 cases). Classifiers are often more sensitive to the majority class and less sensitive to the minority class, leading to biased classification [27]. ADASYN was employed in this study to oversample the minority class (preterm) to balance the term and preterm samples [28]. Therefore, the sample size of PE increased from 19 to 135 cases, and PL increased from 19 to 111 cases. In total, there were 278 cases in PE and TE group, and 230 cases in PL and TL group (Fig. 5).

#### 2.5.2 Random forest

31 features/case☓278 cases from PE and TE group, and 31 features/case☓230 cases from PL and TL group were respectively divided into subset 1 to n and entered to the base learner DT (tree-1, tree-2, …, tree-m) randomly. The value of n was determined by the number of features. The number of features in each subset was chosen randomly but not exceeding the preset maximum. The value of m is the number of base learner DT. The depth d determines the maximum layer each tree can reach.

A DT, which is applied to select features, is formed by randomly selected subset of features. The feature importance is ranked based on its influence on the DT prediction results indicated by out-of-bag (OOB) index. With the ranked features, all DTs in the forest would vote for the most popular class [22].

#### 2.5.3 Classification evaluation

Six-fold cross validation method was applied to evaluate the RF performance for classifying preterm and term delivery, independently for the PE and TE group and for the PL and TL group. The PE and TE group, and the PL and TL group were randomly partitioned into six subsets respectively, five of which were employed to train the RF, the other was used to test the RF. The cross-validation process was repeated six times, with each of the six subsets used once as test data.

The accuracy (ACC), sensitivity, specificity [29] from the six-fold cross validation were averaged to evaluate the performance of RF classification results, independently for the PE and TE group, and for the PL and TL group. The area under the curve (AUC) from the receiver operating characteristic (ROC) curve was also calculated and compared between the PE and TE group, and the PL and TL group.

## 3 Results

### 3.1 Comparison of EHG features between groups of term and preterm delivery

**Fig. 6:**
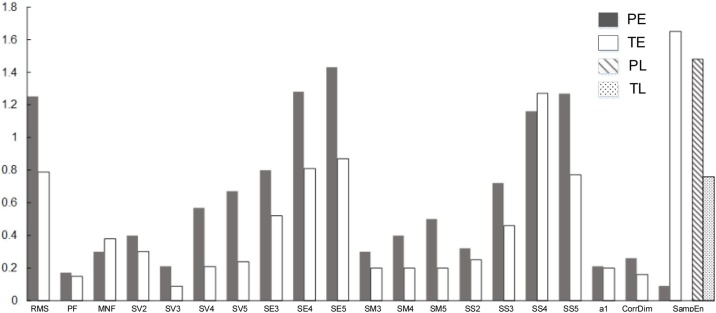
EHG features from PE and TE, PL and TL groups with significant difference in median (p < 0.05).

The 31 EHG features from PE and TE group, PL and TL group are summarized in Table 2. PF, SV2,SV3,SV4,SV5,SE3,SE4,SE5,SM3,SM4,SM5,SS2,SS3,SS4,SS5 of wavelet decomposition, $a_{1}$ of AR model and CorrDim from PE were significantly larger than those of TE (all p < 0.05), while RMS, MNF and SampEn from PE were significantly smaller than TE (all p < 0.05). SampEn of PL was significantly larger than TL (p < 0.05). No other significant difference was found. The features with significant difference are shown in Fig. 6.

**Table 2:** EHG features from PE and TE, PL and TL groups (median (25 %, 75 %)).

	Features	PE	TE	PL	TL
	***Time-related***				
Linear features	RMS[μV]x10	***1.25(0.98,2.14)***a*	0.79(0.56,1.11)	0.75(0.57,1.22)	0.88(0.67,1.09)
	${\text{τ}}_{Rxx}$[s]	36.92(23.15,56.21)	31.42(21.23,58.76)	17.74(9.90,28.62)	28.69(13.72,48.54)
	***Frequency-*** ***related***				
	PF[Hz]	***0.17(0.14,0.19)***a*	0.15(0.13,0.18)	0.16(0.13,0.21)	0.17(0.15,0.19)
	MDF[Hz]	0.19(0.17,0.22)	0.19(0.16,0.23)	0.19(0.16,0.24)	0.21(0.18,0.22)
	MNF[Hz]	0.30(0.27,0.41)	***0.38(0.30,0.50)***a*	0.34(0.28,0.47)	0.35(0.31,0.46)
	***Wavelet-decomposition***				
	SV2×10^−5^	***0.40(0.20,0.90)***a*	0.30(0.20,0.50)	0.20(0.10,0.40)	0.30(0.20,0.60)
	SV3×10^−4^	***0.21(0.11,0.37)***a*	0.09(0.04,0.19)	0.08(0.04,0.23)	0.14(0.06,0.28)
	SV4×10^−4^	***0.57(0.30,1.05)***a*	0.21(0.10,0.45)	0.20(0.10,0.60)	0.35(0.16,0.68)
	SV5×10^−4^	***0.67(0.33,1.43)***a*	0.24(0.12,0.46)	0.25(0.15,0.59)	0.40(0.21,0.83)
	SE2	35.27(25.12,51.24)	28.70(22.90,38.05)	24.96(19.78,35.17)	31.50(22.00,42.10)
	SE3×10^2^	***0.80(0.53,1.04)***a*	0.52(0.34,0.76)	0.47(0.33,0.80)	0.66(0.40,0.88)
	SE4×10^2^	***1.28(0.98,1.67)***a*	0.81(0.57,1.16)	0.68(0.55,1.33)	0.94(0.69,1.35)
	SE5×10^2^	***1.43(1.03, 2.00)***a*	0.87(0.59, 1.21)	0.81(0.66, 1.37)	1.06(0.77, 1.47)
	SM2	0.02(0.01,0.03)	0.01(0.01,0.02)	0.02(0.01,0.02)	0.01(0.01,0.02)
	SM3×10^−1^	***0.30(0.20,0.50)***a*	0.20(0.10,0.30)	0.20(0.20,0.30)	0.20(0.10,0.40)
	SM4×10^−5^	***0.40(0.30,0.70)***a*	0.20(0.20,0.40)	0.30(0.20,0.50)	0.30(0.20,0.50)
	SM5×10^−5^	***0.50(0.30,0.60)***a*	0.20(0.20,0.40)	0.30(0.20,0.40)	0.30(0.20,0.50)
	SS2	***0.32(0.22,0.47)***a*	0.25(0.20,0.36)	0.24(0.18,0.32)	0.29(0.20,0.38)
	SS3	***0.72(0.52,0.94)***a*	0.46(0.31,0.68)	0.44(0.33,0.74)	0.58(0.38,0.82)
	SS4	***1.16(0.84,1.59)***a*	0.72(0.50,1.04)	0.69(0.49,1.20)	0.92(0.62,1.27)
	SS5	***1.27(0.89,1.85)***a*	0.77(0.53,1.05)	0.78(0.60,1.19)	0.98(0.71,1.41)
	***AR-model***				
	$a_{1}\times$10	***0.21(0.20,0.33)***a*	0.20(0.18,0.24)	0.20(0.18,0.21)	0.20(0.18,0.22)
	$a_{2}$	−1.50(−4.82,−1.43)	−1.34(−2.64,−1.07)	−1.34(−1.50,−0.99)	−1.29(−1.99,−1.04)
	$a_{3}$	0.19(−0.06,4.42)	0.06(−0.10,1.52)	0.01(−0.21,0.19)	−0.01(−0.17,0.77)
	$a_{4}$	0.59(−2.40,0.69)	0.55(−0.31,0.65)	0.62(0.42,0.68)	0.53(0.27,0.68)
	$a_{5}$	−0.21(−0.39,0.57)	−0.25(−0.33,0.11)	−0.29(−0.39,−0.13)	−0.27(−0.35,−0.05)
	e x 10^−3^	0.71(0.33,0.91)	0.80(0.48,0.90)	0.80(0.60,0.90)	0.80(0.60,0.90)
Non-linear features	**Tr x 10^−9^**	0.53(0.41,0.66)	0.55(0.41,0.62)	0.56(0.40,0.58)	0.54(0.40,0.70)
	LE	0.20(0.17,0.33)	0.28(0.20,0.43)	0.20(0.09,0.34)	0.25(0.16,0.36)
	SampEn	0.09(0.06,0.11)	***1.65(1.48,1.79)***a*	***1.48(1.24,1.60)***b*	0.76(0.65,0.88)
	CorrDim	***0.26(0.17,0.32)***a*	0.16(0.09,0.22)	0.15(0.11,0.17)	0.17(0.10,0.21)

a* *p < 0.05 between PE and TE*.

b* *p < 0.05 between PL and TL*.

### 3.2 Feature importance

Table 3 shows the 15 key features which were identified as the best features for recognizing preterm delivery both in PE and TE group, and PL and TL group. The feature importance accounted for less than 0.1 % were *a_2_*, SM_3_, SV_3_, SV_4_, SS_3_ in PE and TE group, and *a_2,_ a_3,_* SE_5,_ SM_5,_ SV_4,_ SV_5_ in PL and TL group. It was noticed that SampEn, MDF, MNF, SE4, SM2 and SM4 played important roles on the classification of preterm and term delivery in both PE and TE, PL and TL groups. In particular, SampEn accounted for nearly 70 % of the importance for recognizing preterm delivery.

**Table 3:** Feature importance for recognizing preterm delivery, separately for PE and TE, and PL and TL groups.

Features	PE and TE group	PL and TL group
SampEn	66.67 %	69.53 %
MDF	9.34 %	1.32 %
MNF	3.67 %	7.53 %
*a_2_*	<0.1 %	<0.1 %
*a_3_*	3.67 %	<0.1 %
SE_4_	1.13 %	0.43 %
SE_5_	3.67 %	<0.1 %
SM_2_	2.26 %	4.73 %
SM_3_	<0.1 %	6.17 %
SM_4_	3.67 %	4.35 %
SM_5_	2.26 %	<0.1 %
SV_3_	<0.1 %	5.00 %
SV_4_	<0.1 %	<0.1 %
SV_5_	3.26 %	<0.1 %
SS_3_	<0.1 %	0.44 %

### 3.3 Evaluation of RF classifier

**Fig. 7:**
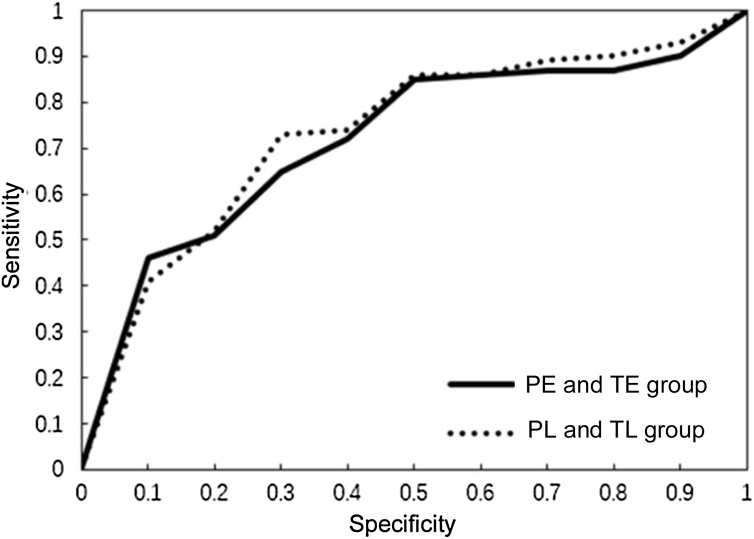
ROC curve for classification of PE and TE, PL and TL.

ROC curves for classifying preterm delivery in PE and TE group, and PL and TL group are shown in Fig. 7. There was no significant difference between the two AUCs from the ROC curves (p = 0.70). As shown in Table 4, RF achieved the ACC of 0.92, sensitivity of 0.88, specificity of 0.96 and AUC of 0.88 for PE and TE group, and ACC of 0.93, sensitivity of 0.89, specificity of 0.97, and AUC of 0.80 for PL and TL group.

**Table 4:** Evaluation of RF classifier and summary of research work on prediction of preterm delivery using the same TPEHG database.

Author	Classifier	Data	Accuracy	Sensitivity	Specificity	AUC	Chosen signals	Feature (number)
Current study	RF	PE and TE	0.92	0.88	0.96	0.88	0.08−4 Hz, the remaining 20 min length of signal	RMS, PF, SampEn, CorrDim, etc (31)
		PL and TL	0.93	0.89	0.97	0.80		
Naeem et al. [8]	Trainable cascade-forward network	Preterm and term	0.85				0.3−3 Hz, the whole 30 min length of signal	RMS, ZC, PF, approximate entropy,etc (11)
								
Smrdel et al. [9]	Adaptive autoregressive	PE and TE	0.97	1.00	0.95		0.34−1 Hz, 0.3−4 Hz, the whole 30 min length of signal	SampEn, MDF (2)
		PL and TL	1.00	1.00	1.00			
								
Ahmed et al. [11]	MMFE and MMSE algorithms	PE and TE	0.95	0.92	0.98	0.99	0.3−3 Hz, the remaining 27 min length of signal	SampEn (1)
								
Fergus et al. [16]	Advanced artificial neural network	Preterm and term	0.88	0.91	0.84	0.94	0.34−1 Hz, the whole 30 min length of signal	RMS, PF, MDF, maximum fractal length, etc (10)
								
Ren et al. [17]	Adaboost	Preterm and term				0.99	0.3−3 Hz, the whole 30 min length of signal	RMS, SampEn, Shannon entropy ratios (5)
								
Fergus et al. [18]	Linear discriminant classifier	Preterm and term	0.93	0.97	0.90	0.97	0.34−1 Hz, the whole 30 min length of signal	RMS, PF, MDF, SampEn (4)
	Polynomial classifier		0.93	0.97	0.90	0.95		
	Logistic classifier		0.93	0.97	0.90	0.94		
	Decision tree		0.93	0.97	0.90	0.93		
								
Idowu et al. [19]	RF, penalized logistic regression and rule-based classifier	Preterm and term	0.91	0.97	0.85	0.94	0.34−1 Hz, the whole 30 min length of signal	RMS, PF, MDF, SampEn(4)
								
Acharya et al. [21]	SVM	Preterm and term	0.96	0.95	0.97	0.96	0.3−3 Hz, the remaining 24 min length of signal	intrinsic mode functions (8)
								
Jager et al. [28]	QDA	PE and TE	1.00	1.00	1.00	1.00	0.08−5 Hz, the whole 30 min length of signal	*PA, MDF, SampEn (11)
		Preterm and term	0.96	0.94	0.98	0.99		

*PA, peak amplitude of the normalized power spectrum. 11 features (PA, MDF, SampEn) selected from 5 bands, respectively.

Table 4 summarizes the performance of RF model in this study in terms of ACC, sensitivity, specificity and AUC, in comparison with the previously published papers using TPEHG database [8,9,11,[16], [17], [18], [19],^21^,^28^]. All the studies achieved over 80 % ACC and sensitivity.

## 4 Discussion

In this study, RF classifiers were developed using EHG signals recorded before and after the 26^th^ gestational week to recognize the preterm delivery. Among the extracted EHG features, SampEn, MDF, MNF, SE4, SM2 and SM4 were more important for classification of preterm and term delivery whether early or later recorded. With RF classifier, the classification results in PE and TE group (ACC of 0.92, SE of 0.88, SP of 0.96, AUC of 0.88) were similar to the results in PL and TL group (ACC of 0.93, SE of 0.89, SP of 0.97, AUC of 0.80).

Compared with other studies using TPEHG database, the current study extracted EHG features including 27 linear and 4 nonlinear features more comprehensively. RF classifier which did not require computational complexity, performed a promising result without additional step of pre-selected features in a wider band pass filter of 0.08−4 Hz. The feature importance was ranked by RF based on classification accuracy. After the importance of different features was ranked by DT, SampEn was found to be the most important feature for recognizing preterm delivery. The previous studies concluded that nonlinear methods such as sample entropy [9,20], approximate entropy [8,20] and Shannon entropy [17] can provide better discrimination between pregnancy and labor contractions compared to linear methods [34]. It is probably because entropy reflects the complex and nonlinear dynamic interactions between myometrium cells [8,23]. SampEn was considered to be particularly suitable for revealing EHG changes in relation to pregnancy progression and labor [33]. RF classifier could obtain the promising results as the previous studies illustrated [17,19].

The performance of recognizing preterm delivery was influenced by the cut-off frequency of filter and the extracted features. Jager et al. [28] got the highest classification ACC of 100 % with features from the frequency band of 0.08∼5 Hz when using the entire records of TPEHG database. Most of studies used the specific features [9,11,30] or selected features [8,[16], [17], [18],^21^] for prediction of preterm delivery, while RF utilized the extracted features without additional feature selection algorithm. Similar to the other studies in Table 4, the current study extracted features from the entire records because there were no annotated contraction intervals or even no contraction during early recordings. Recently, various features and classifiers have been proposed to recognize uterine contraction (UC) with Icelandic 16-electrode database [20,[31], [32], [33]]. As UC detection is necessary for monitoring labor progress, some studies extracted features from EHG bursts [20,32,33] and achieved reliable results of UC detection by machine learning and deep learning algorithms [[35], [36], [37]]. A multi-channel system for recognizing uterine activity with EHG signal has also been developed in clinical research [38]. They also provided important ways for recognition of preterm delivery with UC.

ADASYN technique was applied to solve the problem of unbalanced data in our study, though synthetic minority oversampling technique (SMOTE) algorithm has been employed in the previous studies [[16], [17], [18],^23^]. Compared with ADASYN technique, the synthetic samples generated by SMOTE algorithm may increase the likelihood of data overlapping which will not provide more useful information [12,27]. ADASYN achieved better results for classification of preterm delivery in current study.

The present work has the following limitations. The synthetic data generated by ADASYN is less convincing than the clinically collected EHG data. More clinical EHG signals are essential, in particular from preterm delivery. A comprehensive study has been conducted on various EHG features, however, sixteen of which were from wavelet decomposition coefficients. Therefore, AAR model [9], EMD technique [17], multivariate multiscale entropy features [8] and combination of multi-channel EHG signals [5,11,39] could be investigated to improve the prediction of preterm delivery [39]. Nevertheless, as a pilot study, the positive results from using channel 3 was the first step for evaluating the effectiveness of a RF model. Furthermore, comparison of different classifiers for recognizing preterm delivery could be considered in future study.

## 5 Conclusion

In current study, sample entropy played the most important role on recognizing preterm delivery among the 31 extracted features. RF classifier was a promising method without additional steps of selecting features. EHG signals recorded before the 26^th^ week of gestation achieved the similar results to those after the 26^th^ week. This study is of great helpful in the early prediction of preterm delivery and early clinical intervention.

## Authors’ contributions

Jin Peng, Hongqing Jiang designed the analysis method and classifiers; Lin Yang and Mengqing Du assisted with signal preprocessing; Xiaoxiao Song and Yunhan Zhang assisted with data curation; Jin Peng analyzed the results and wrote the original draft; Dongmei Hao and Dingchang Zheng reviewed the draft

## Ethics approval and consent to participate

The analysis of this database was approved by the Research Ethics Committee of the Faculty Research Ethics Panel (FREP) of Beijing University of Technology and Anglia Ruskin University.

## Availability of data and materials

The database used in this study is available to access via the link: http://lbcsi.fri.uni-lj.si/tpehgdb/ or https://www.physionet.org/physiobank/database/tpehgdb/.
